# Noninvasive Prediction of Advanced Fibrosis in Pediatric Liver Disease—Discriminatory Performance of 2D Shear Wave Elastography, Transient Elastography and Magnetic Resonance Elastography in Comparison to Histopathology

**DOI:** 10.3390/diagnostics12112785

**Published:** 2022-11-14

**Authors:** Jon Nielsen, Mette Skalshøi Kjær, Allan Rasmussen, Deepthi Chiranth, Gro Linno Willemoe, Birthe Merete Henriksen, Lotte Borgwardt, Mia Klinten Grand, Lise Borgwardt, Vibeke Brix Christensen

**Affiliations:** 1 Department of Paediatrics and Adolescent Medicine, Copenhagen University Hospital, Rigshospitalet, Blegdamsvej 9, 2100 Copenhagen, Denmark; 2 Department of Medical Gastroenterology and Hepatology, Copenhagen University Hospital, Rigshospitalet, Blegdamsvej 9, 2100 Copenhagen, Denmark; 3 Department of Surgical Gastroenterology and Transplantation, Copenhagen University Hospital, Rigshospitalet, Blegdamsvej 9, 2100 Copenhagen, Denmark; 4 Department of Pathology, Copenhagen University Hospital, Rigshospitalet, Blegdamsvej 9, 2100 Copenhagen, Denmark; 5 Department of Diagnostic Radiology, Copenhagen University Hospital, Rigshospitalet, Blegdamsvej 9, 2100 Copenhagen, Denmark; 6 Department of Biostatistics, Faculty of Health Sciences, Institute of Public Health, University of Copenhagen, Blegdamsvej 3B, 2200 Copenhagen, Denmark; 7 Department of Clinical Physiology, Nuclear Medicine and PET, Copenhagen University Hospital, Rigshospitalet, Blegdamsvej 9, 2100 Copenhagen, Denmark

**Keywords:** liver fibrosis, elastography, pediatric liver disease, ultrasound elastography, 2D shear wave elastography, transient elastography, magnetic resonance elastography

## Abstract

*Background*: Elastography can be measured with different imaging techniques and is increasingly used for noninvasive assessment of hepatic fibrosis. Little is known about the performance, and interrelation of different elastographic techniques, in prediction of hepatic fibrosis in pediatric liver disease. *Objectives*: We aimed to determine the discriminatory value for advanced fibrosis (Metavir F3-4) and evaluate the applicability of 2D shear wave ultrasound elastography (USe), Transient Elastography (TE) and Magnetic Resonance elastography (MRe) in pediatric liver disease. *Methods:* In patients with pediatric liver disease aged 0–19 years, USe, TE and MRe were compared with histopathological fibrosis stage. Multivariate logistic regression models for advanced fibrosis were considered. Discriminative performance was assessed by the area under the receiver operating characteristic curve and the Brier Score. Primary analyses included complete cases. Multiple imputation was used as sensitivity analysis. *Results*: In 93 histologically evaluated patients USe, TE and MRe were performed 89, 93 and 61 times respectively. With increased liver stiffness values, significantly increased odds for presenting F3-4 were seen in individual models for ALT < 470 U/L, whereas the effect for ALT > 470 U/L was non-significant. Area under the curve and Brier Score for discrimination of advanced fibrosis were 0.798 (0.661–0.935) and 0.115 (0.064–0.166); 0.862 (0.758–0.966) and 0.118 (0.065–0.171); 0.896 (0.798–0.994) and 0.098 (0.049–0.148) for USe, TE and MRe respectively. No significant increase in discriminatory ability was found when combining elastographic modalities. *Conclusions*: In pediatric liver disease, USe, TE and MRe had a good discriminatory ability for assessment of advanced liver fibrosis, although TE and MRe performed best. In most children with pediatric liver disease, TE is a reliable and easily applicable measure.

## 1. Introduction

Liver fibrosis of varying degrees is the final outcome across disease categories in pediatric liver disease. The severity of fibrosis is commonly used to guide treatment options, and evaluate disease status [1,2]. Consequently, reliable measures of fibrosis are crucial for physicians in the field of pediatric hepatology.

Though liver biopsy remains the gold standard for assessment of fibrosis stage [3], some inherent problems are associated with its use, especially in children. Pediatric liver biopsy is largely a safe procedure in the hands of an experienced operator. However, it is an invasive procedure requiring general anesthesia, and it carries a risk of major and minor bleeding, along with frequent abdominal pain [4]. Moreover histologically, the sparse proportion of total liver tissue has been reported to be subject to sampling variability, interobserver variability and dependence on core number and -length for proper staging of fibrosis [5].

Noninvasive methods for assessing liver fibrosis are internationally demanded and persistently sought. In recent years, several noninvasive methods for assessment of fibrosis have become available. The propagation speed of shear waves through the tissue as a measure of liver stiffness has become an area of interest. After induction of tissue stress, 2D shear wave ultrasound elastography (USe), Transient Elastography (TE) and Magnetic Resonance elastography (MRe) all measure shear waves propagating through the tissue. A reduction in the hepatic elasticity, as a consequence of pathological processes including fibrotic transformation, leads to faster propagation speed, and a quantitative liver stiffness measure can be obtained [6,7]. Whereas TE primarily provides a stiffness value, USe and MRe are both obtained as part of an imaging procedure and hence other features are often interpreted in parallel with the elastography values.

The performances of USe, TE and MRe for distinction of histopathological fibrosis stages have primarily been investigated in adult liver disease, and all methods have been reported to possess high accuracies for advanced fibrosis, whereas discrimination of lower fibrosis stages are less accurate upon pooled analyses [8]. At present, less is known about the discriminatory performance of elastography in pediatric liver disease. The performance of USe and TE for predicting fibrosis have been investigated sporadically in smaller pediatric cohorts, and cirrhosis reported to be accurately predicted, whereas distinction between other fibrosis stages were less accurate [9,10,11]. A high success rate of pediatric MRe has been reported [12] and a few studies, largely focusing on nonalcoholic fatty liver disease and nonalcoholic steatohepatitis, have reported on the relation between pediatric MRe results and histopathological fibrosis stage [13,14,15].

Patient characteristics and the spectrum of liver diseases are markedly different in the pediatric population compared to adults, thus results from adult studies do not apply to pediatric patients. The discriminatory performance of all three elastographic methods in the same cohort of patients with pediatric liver disease are yet to be evaluated. More knowledge is needed to clarify the use of each elastographic measure in children and adolescents with a range of liver diseases, and the effect of combining different elastographic modalities. In a cohort of patients with pediatric liver disease of various etiologies, we aimed to evaluate feasibility and diagnostic accuracy for predicting advanced histopathological fibrosis stage using USe, TE and MRe respectively. We hypothesized the three modalities to possess comparable accuracies for predicting advanced fibrosis in these children.

## 2. Material and Methods

### 2.1. Patients

Pediatric patients aged 0–19 years of age with known chronic or acute liver disease, suspected liver disease (elevated transaminases of unknown origin), or children with prior liver transplantation, in which liver biopsy and either USe, TE or MRe, or a combination of the latter three, had been performed between December 2015 and March 2020, were included in this study. All patients were part of prospective observational cohort and all eligible patients fulfilling the above criteria were included. Elastographic measures were included for comparison, if they were performed within a maximum of 3 months from liver biopsy. MRe was not performed in children aged less than 5 years, due to the need for general anesthesia in these patients. Children with neoplasms/malignant liver disease or children with severe instability hindering examinations were excluded. Informed consent from the holders of custody/patient was obtained on all included patients.

### 2.2. Histopathology

Through ultrasound guided intercostal access two liver biopsies (2 cm/18 G) were obtained from the right hepatic lobe in the region intended for elastographic measures. In patients with split liver grafts, biopsies were typically obtained through subcostal access. Biopsies were fixed in formalin, paraffin embedded and cut in 2–4 µm thick sections for staining. Routine stains included haematoxylin/eosin at 4 levels interspersed in between the special stains, which include Periodic Acid-Schiff, Periodic Acid-Schiff with diastase, Masson’s trichrome, modified Sirius, oxidised orcein, Perl’s stain for iron and reticulin Artisan.

All biopsies were reviewed by two experienced hepatopathologists, blinded to patient diagnosis, and to the results from the other. Biopsies were considered suitable for analysis, if they contained at least 11 portal tracts. Fibrosis was graded by a combined assessment of features on haematoxylin/eosin, Masson’s trichrome, modified Sirius and oxidised orcein stains. It was considered present only when elastin fiber deposition was noted on oxidised orcein stain [16,17]. Fibrosis staging was performed according to the Metavir scoring system on a scale of 0–4 as follows: F0; no fibrosis, F1; portal fibrosis without septa, F2; portal fibrosis with rare septa, F3; numerous septa without cirrhosis, F4; cirrhosis [18]. F3 or higher defined advanced fibrosis. Fibrosis scores by the two hepatopathologists were compared for concordance. In cases of discordance, differences in scoring were discussed, and consensus achieved.

Inflammation was scored semiquantitatively (mild/moderate/severe). Necrosis was categorised as single-cell/confluent with collapse. Canalicular/cellular cholestasis was noted, and venous outflow obstruction considered present if central veins and sinusoids were dilated or showed signs of congestion.

### 2.3. Ultrasound Elastography

USe was performed as 2D shear wave elastography [19,20] using LOGIQ E9 machines with a curved C1-6VN transducer (General Electric Healthcare, Chicago, IL, USA). USe was performed together with a standard B-mode examination of the liver and spleen including flow evaluation. By protocol USe was performed in the right hepatic lobe through intercostal access, with the patient in the supine position, and the right arm elevated. In patients with prior transplantation, the liver graft was mostly only visualizable through subcostal access and the USe was performed from this location. The top of the shear wave box was positioned 1–2 cm below the liver capsule with the middle of the box between 3–5 cm, avoiding visible vessels. Typically time spent on USe examination was 15 min (about half the time spent on obtaining elastographic measures and performing calculations). If USe was performed together with liver biopsy, measurements were obtained prior to biopsy, in the region intended for tissue sampling. Patients in general anesthesia fasted for six hours, while other patients fasted for four hours prior to examination. In smaller children however, two hours of fasting was accepted if four hours was not achievable. Measurements were performed in apnea if possible, to reduce artifacts. Examinations were performed by six unblinded radiologists, all with more than 5 years of experience with pediatric ultrasound and USe. Minimum ten acquisitions per examination were obtained. Blinded to diagnosis, one experienced radiologist evaluated the measurements and did the calculations. According to guidance from the machine manufactor acquisitions were excluded if the colorbox was not uniformly filled-in for at least 50%. Acquisitions with movement artifacts were excluded. Calculations were obtained from a 1 cm region of interest, avoiding vessels and obvious artifacts. The USe value is the median of 10 valid calculations. Values with interquartile range/median of >0.15 were excluded according to guidelines and recommendations from the World Federation for Ultrasound in Medicine and Biology [20].

### 2.4. Transient Elastography

TE was measured using the FibroScan^®^ device (Echosens^®^, Paris, France). After an induction pulse, the shear wave velocity through a hepatic tissue cylinder (approximately 1 × 4 cm) is converted to a stiffness measure (kPa), and returned as the median value of the successful measures [21,22]. Four unblinded experienced operators performed the measures, and patients fasted for four hours prior to examination. In smaller children however, two hours of fasting was accepted if four hours was not achievable. With the patient in the supine position and right arm elevated, the transducer was placed over the right hepatic lobe in the 9th to 11th right intercostal space. In transplanted patients either the epigastric region or the right intercostal space was used. The S1, S2 and M-probe were used when the patient thoracic circumferences where ≤45 cm, 45–75 cm and ≥75 cm respectively (<15 kg, between 15–35 kg and above 35 kg were recommended for S1, S2 and M if circumference was not measured). Typical examination time was between 5–10 min. As recommended by the manufactor, we aimed for 10 valid readings with an interquartile range <30% of the median, and a success rate above 60%. These original criteria was explored further by Boursier et al. concluding that interquartile range/median below 30% was only important if TE measurement exceed 7.1 kPa and success rate was abandoned [23]. Further evidence suggests that three to five valid measurements provide the same diagnostic accuracy [24]. Hence patients with a minimum of 5 valid TE measures, and interquartile range/Median ≤ 30% if the result was above 7.1 kPa were included for analyses.

### 2.5. Magnetic Resonance Elastography

MRe was performed on a 1.5-T MR scanner (GE Optima™ 450W, General Electric Healthcare, Chicago, IL, USA). Active driver produced vibrations at 60 Hz were transmitted to a passive driver placed over the right hepatic lobe. Shear waves were imaged with a modified phase-contrast gradient echo sequence (slice thickness 8 mm; gap 8 mm; matrix 256 × 64; repetition time/echo time 50/21.9 ms; bandwidth ± 31.25 kHz; flip angle 30; field of view 32–42 cm), and elastograms were generated. Patients fasted for four hours prior to examination. The examination, including MR cholangiopancreatography and MRe, lasted for about 60 min. Though MR cholangiopancreatography was performed, other MR imaging features were not assessed when performing the elastographic measurements and such features were solely used for guiding the placement of the region of interest. Accordingly, the MRe values were solely the result of standard calculation of kPa values and not influenced by other features seen on the MR examination. Since description and evaluation of other features and structures on the MR examination was not part of the study, these features were assessed separately at a later point in time and hence not included in the present study. Blinded to diagnosis, one experienced MR radiologist processed all examinations and performed the measurements. Three MRe slices were obtained through the largest cross-section, avoiding the dome and the inferior part of the liver. Avoiding liver edges, fissures, large vessels, wave interferences and artifacts, the largest possible region of interest was drawn based on the magnitude image as reference. Elastography values were computed for each slice, and overall stiffness values for the whole liver were recorded as the mean of all slices. The quality of the MRe was judged by interpretation of the wave image. We aimed for wave propagation forming parallel lines through the liver. If this was not obtained and a disrupted wave pattern was found, the driver was repositioned over the liver to optimize the image.

### 2.6. Statistical Analyses

Categorical data are presented as counts and percentages. Continuous data are presented as median with interquartile range or range. Failure rates were compared using Fisher’s exact test.

To identify the clinically most accurate and applicable model for predicting advanced fibrosis (F ≥ 3–4), five multivariate logistic regression models were considered. Outcome was advanced fibrosis, and age, Alanine aminotransferase level together with either one or all three elastographic measures or the combination of the two models presenting the highest discriminatory values in individual models, were included as covariates. Age and elastographic measures were included as continous variables whereas Alanine aminotransferase > 470 U/L (10× upper reference limit) was included as a binary variable. Results were reported as odds ratios with 95% confidence intervals.

The primary analyses included complete cases only. To assess a potential effect of missing data in the covariates (USe, TE and MRe), multiple imputation was performed as a sensitivity analysis [25]. The models for the outcome were the same in complete case and multiple imputation analyses. Multiple imputation models included fibrosis stage, USe, TE, MRe, alanine aminotransferase, age, sex, prior transplantation, body mass index, bilirubin, thrombocytes and international normalised ratio. The total number of imputations was 20. Imputations were performed on log-transformed data to ensure valid imputation.

Multiple observations in the same subjects were considered dependent if no liver transplantation was performed between two consecutive measures, whereas observations were considered independent if transplantation was performed between two consecutive measures. To account for dependence, the robust sandwich-estimator with working independence was used for the variance in complete case analyses, and bootstrapped confidence intervals based on 1000 samples using five imputations were used for multiple imputation analyses [26].

Patients aged less than five years were removed before analysis if the model included MRe.

The ability of the models to discriminate advanced fibrosis was assessed using receiver operating characteristic curves and area under the curve. Accuracy and calibration of the models was evaluated using Brier Score. Since MRe only included patients aged ≥ 5 years, discriminatory ability and calibration of USe and TE were evaluated both as values for all ages and for those aged ≥ 5 years only.

Nomograms for the risk of advanced fibrosis was constructed for the final models, and based on Youden’s index, the cut-off value presenting a specificity of 90% was identified.

Statistical analyses were performed using R 3.6.0 (R Core-Team 2019) package smcfcs (J. Bartlett/R. Keogh, 2019) and SAS^®^ version 9.4 for Windows (SAS Institute Inc., Cary, NC, USA). Two-sided *p*-values < 0.05 were considered statistically significant.

## 3. Results

Between December 2015 and March 2020, a total of 117 liver biopsies were performed in 93 included patients. Seventeen patients had a repeated liver biopsy while two patients and one patient respectively had biopsy performed thrice and four times. USe, TE and MRe were performed 89, 93 and 61 times respectively and failure rates were 14.6% (13/89), 15.1% (14/93) and 1.6% (1/61). Causes of failure were: restless patient (9/13), failure to save data in machine (2/13), obesity (1/13) and too few measurements (1/13) for USe; high interquartile range/median (13/14) and too few measurements (1/14) for TE; failure to generate elastogram based on non-optimal placement of the transducer (right sided transducer placement in a patient with a centrally located liver graft) (1/1) for MRe. Failure rate for MRe was significantly lower compared to USe and TE (*p* = 0.01). The median (range) days between biopsy and elastography were 0 (0–85 days), 1 (0–90 days) and 1 (0–90 days) for USe, TE and MRe respectively. Seven patients had less than 10 TE measures performed (five measures in four patients and six to nine measures in three patients) while the rest had at least ten measures performed.

An equal distribution of sex was found in included patients. Autoimmune hepatitis, former liver transplantation and biliary atresia were the most common causes for inclusion (29.1%, 26.9% and 10.8% of included patients respectively). Characteristics of included patients are found in Table 1.

The distribution of fibrosis stages in included biopsies were F0; 20.5% (24/117), F1; 23.9% (28/117), F2; 29.1% (34/117), F3; 7.7% (9/117) and F4; 18.8% (22/117). Agreement in fibrosis scoring between the hepatopathologists was 100% for cirrhosis. Disagreement was seen in 11.1% (13/117) of the biopsies (7/13 between F1–F2, 3/13 between F0–F1 and 3/13 between F2/F3). 92.3% (12/13) of the biopsies with discordance were eventually scored as ≤F2.

**Figure 1:**
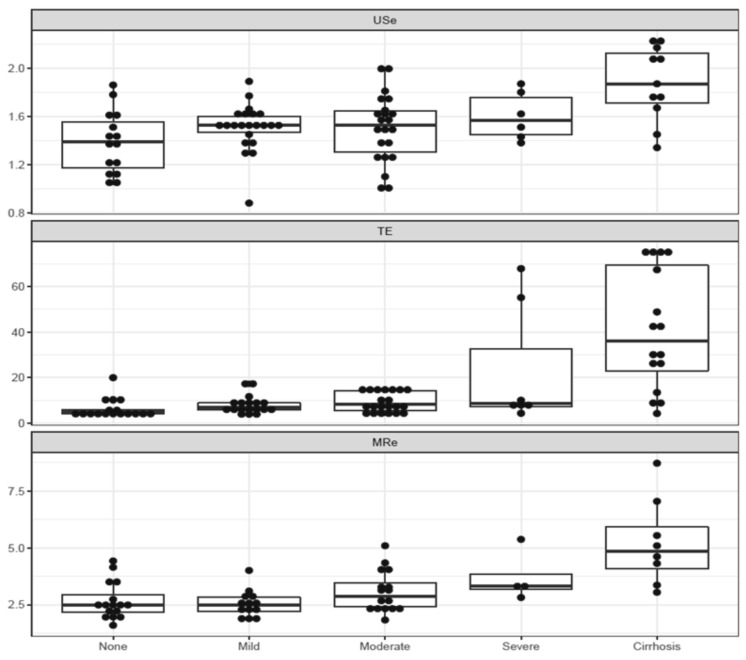
Box- and dotplots of USe, TE and MRe in each fibrosis stage, showing the median (line in box) and interquartile range (IQR, box lines). Upper and lower whiskers represent Q3 + 1.5 × IQR respectively Q1–1.5 × IQR. X-axis: fibrosis stage. Y-axis: Liver stiffness measure (m/s for USe and kPa for TE and MRe).

Liver stiffness (median (interquartile range)) for F0–F2 compared to F3–F4 were 1.52 m/s (1.33–1.62) vs. 1.77 m/s (1.51–2.07), 6.30 kPa (4.78–9.93) vs. 29.90 kPa (8.90–61.20) and 2.48 kPa (2.22–3.21) vs. 4.64 kPa (3.18–5.47) for USe, TE and MRe respectively. Stiffness values generally increased with increasing fibrosis stage in all three modalities, however substantial overlap was seen, most evident in lower fibrotic stages (Figure 1).

### 3.1. Complete Case Analyses

**Figure 2:**
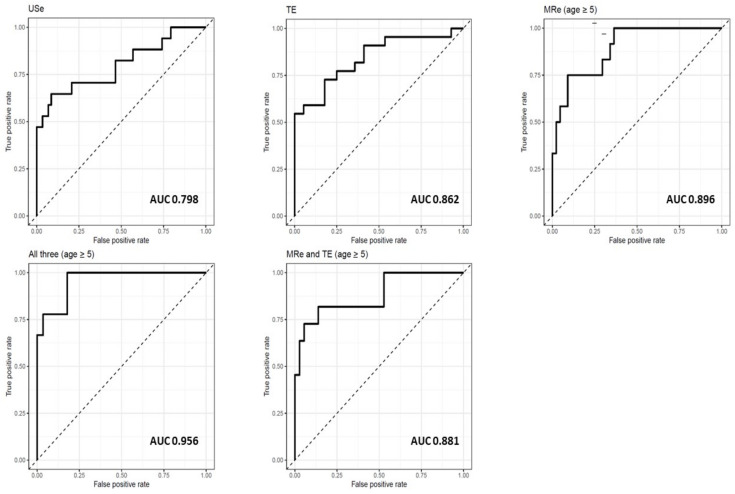
Receiver operating characteristics curves for the complete case analyses.

With increasing elastography values, significantly increased odds for presenting advanced fibrosis were evident (Table 2). For alanine aminotransferase ≤ 470 U/L a one unit increase in USe, a doubling of TE or a doubling of MRe increased the odds for advanced fibrosis 602 times, 5 times and 233 times respectively. The uncertainty of the estimated effect was greater for MRe and, in particular USe, compared to TE. All modalities performed well in distinguishing advanced fibrosis from lower stages, with the highest area under the curve values (0.896 and 0.862) found for MRe and TE, as opposed to poorer performance of USe (0.798) (Figure 2, Table 2). MRe presented the lowest Brier Score compared to USe and TE (Table 2).

When comparing discriminatory abilities of USe and TE, only for patients aged ≥ 5 years, the results were similar with areas under the curve of 0.784 (USe); 0.878 (TE) and Brier Scores of 0.121 (USe); 0.104 (TE) (Table 2).

In models including all three modalities and TE combined with MRe respectively, no significant effect estimates were identified. However, area under the curve marginally increased and Brier Score marginally decreased upon inclusion of all three modalities (Figure 2, Table 3).

The effect of liver stiffness values for alanine aminotransferase > 470 U/L was non-significant in all included models (0.14 (0.01–3.43); 1.15 (0.14–9.67); 2.13 (0.06–70.6) (odds ratio (confidence interval) for USe, TE and MRe)).

### 3.2. Multiple Imputation Analyses

**Figure 3:**
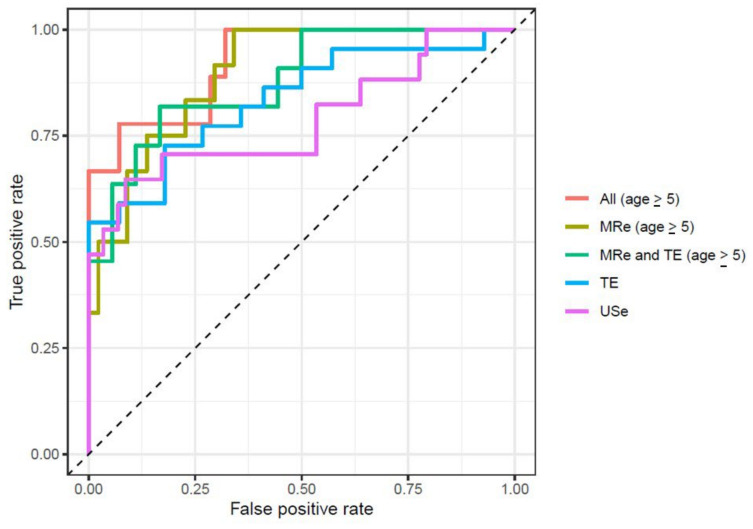
Receiver operating characteristics curves for the multiple imputation models.

Effect estimates and discriminatory values for advanced fibrosis were comparable to the complete case models for all three modalities and discriminatory abilities of USe and TE for those aged ≥ 5 years were comparable to those obtained from patients of all ages (Table 2). The odds for presenting advanced fibrosis increased with elastography measures in all multiple imputation models. As for the complete case models, MRe and TE presented the best performances for diagnosing advanced fibrosis. Receiver operating characteristic curves for all imputation models are found in Figure 3.

As for the complete case analyses, models including more than one modality identified no significant effect estimates but resulted in slightly better discrimination of advanced fibrosis upon inclusion of all three modalities (Table 3). The effect of elastography values for alanine aminotransferase >470 U/L was non-significant in all included multiple imputation models (data not shown).

**Figure 4:**
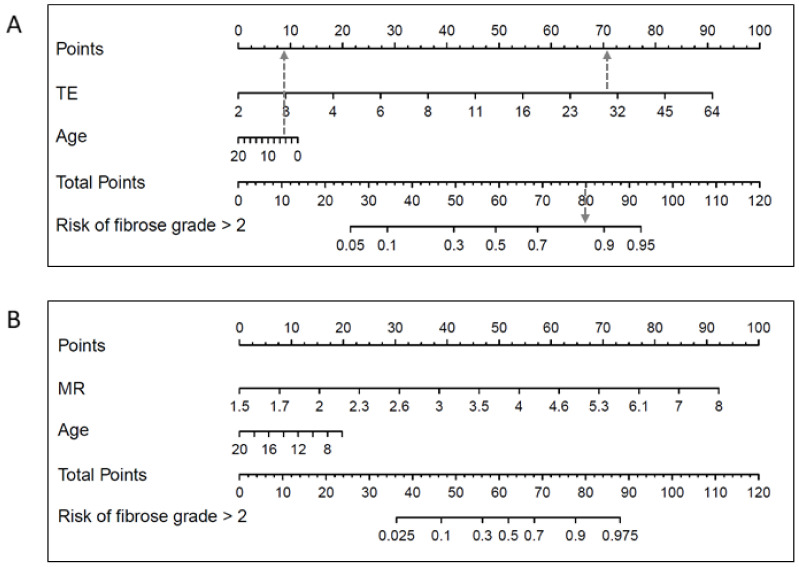
Nomograms for the prediction of advanced fibrosis (F3-4) based on TE results (**A**) and MRe results (**B**) in patients with pediatric liver disease. Grey dotted line: The risk of advanced fibrosis for a 4 year old child with TE result of 30 kPa is almost 0.9. TE: Transient elastography. MRe: Magnetic Resonance elastography.

TE and age along with MRe and age were included in the two final models for predicting advanced fibrosis in patients with pediatric liver disease and alanine aminotransferase ≤470 U/L. Nomograms for these models are presented in Figure 4. For the TE model, a specificity of 0.90 provided a cut-off value of 0.38, a sensitivity of 0.55 and a positive predictive value and negative predictive value of 0.67 and 0.83 respectively, whereas the highest Youden’s index was found for a cut-off value of 0.179 (sensitivity 0.86, specificity 0.70, positive predictive value 0.53 and negative predictive value 0.93). For the MRe model, a specificity of 0.90 provided a cut-off value of 0.33, a sensitivity of 0.75 and a positive predictive value and negative predictive value of 0.69 and 0.93 respectively, whereas the highest Youden’s Index was found for a cut-off of 0.118 (sensitivity 1, specificity 0.66, positive predictive value 0.44 and negative predictive value 1).

## 4. Discussion

In a pediatric cohort covering a variety of liver disease diagnoses, we aimed to compare the ability of three widely used noninvasive elastographic methods, to predict histologically advanced fibrosis. All methods performed well in predicting advanced fibrosis. Overall MRe and TE performed best with areas under the curve of 0.896 and 0.862 and the lowest Brier Score found in MRe whereas Brier Scores for TE and USe were comparable. These results indicate a higher discriminatory ability for advanced fibrosis for MRe and TE compared to USe. In all modalities, the odds for presenting advanced fibrosis significantly increased with elastography value. Inherent differences exist between the three elastography techniques in terms of mode of applied force (ultrasound induced (USe); mechanically induced (TE); driver induced (MRe)), region of interest (narrowest region of interest for USe and largest for MRe), proportion of liver tissue evaluated and depth of measurents among others. Due to these differences the three modalities show markedly different ranges of possible values, which hinders direct comparison of effect estimates, whereas discriminatory values of the models are readily compared [19,22].

Technical advances and a continuous and rational focus on noninvasive prediction of fibrosis, have increased the focus on elastography as a tool for assessment of disease status and progression. As a reflection of obvious benefits of noninvasive methods in pediatric patients, elastography measures are already widely applied in many institutions, as a supplementary decision tool in the clinical evaluation of children with liver disease. However, the clinical relevance and accuracy for predicting histological fibrosis is still not elucidated [22], and liver biopsy remains a central part of disease evaluation in pediatric patients [27]. Especially performance of USe, TE and MRe in the same pediatric patients has not been investigated previously.

The discrimination of USe in staging of hepatic fibrosis has been assessed in smaller studies of pediatric liver disease, with abilities for discriminating advanced fibrosis in various liver diseases, ranging from 0.81 to 0.97 [28,29,30]. Likewise performance of TE for predicting advanced fibrosis in pediatric patients, has been investigated in smaller cohorts, and discriminatory ability reported to range from 0.79 to 0.86 [31,32,33], although values of 1 have been reported for advanced fibrosis [34]. Recent studies of pediatric MRe presented good to excellent discrimination for both significant- and advanced fibrosis (area under the curve of 0.92 and 0.88–0.93 respectively [13,14,15].

Largely, this study confirms the findings of high discriminatory values for predicting advanced fibrosis in pediatric liver disease. To support the conclusive value of our findings, we tested all modalities in the same cohort of patients. The individual models for TE and MRe had high and comparable discriminatory values, however TE displayed less variance and proved to be robust, since results were roughly the same in the multiple imputation analysis, whereas the MRe model was more sensitive. In contrast to adult studies, we did not find a markedly higher discriminatory value of MRe compared to TE [35]. As in adult studies, prior focus of pediatric MRe studies has been on patients with nonalcoholic fatty liver disease/nonalcoholic steatohepatitis, comprising 100%, 77% and 56% of included patients in pediatric studies respectively [13,14,15]. The proportion of children with advanced fibrosis in these MRe studies was lower compared to our cohort, however in our cohort, presenting a range of etiologies, we found performance measures of MRe to be comparable to those obtained in previous pediatric studies. The discriminatory value was poorer for the USe model compared to the other modalities albeit not significant. As for MRe, the effect estimate of USe displayed larger variance, and was less robust, compared to TE. Quality criteria for interpretation of USe and MRe results are still less well defined compared to TE [22] Hence differences in such quality criteria might have affected the observed differences. In addition, TE examinations were performed by physicians familiar to the child, in well-known surroundings, whereas six different radiologists performed the USe exams and, although all were experienced, this heterogeneity may in part explain the lower performance measures of USe.

Close collaboration and knowledge sharing between clinicians and radiologists are of upmost importance upon interpretation of elastographic measures. The radiologists possess valuable information on the quality criteria of the elastography (USe or MRe) and for the clinician it is essential to know whether a certain measure should be trusted or whether it should be interpreted with reservations. When evaluating elastographic measures, importantly the number of measures, the interquartile range and the quality of the imaging needs to be addressed. In the present study all USe or MRe results not fulfilling quality criteria were labeled as a failed measure and likewise TE measures performed by the clinician were also labeled as failed when not fulfilling quality criteria.

A potential age effect on elastography is still not fully clarified. Minor age dependent increases in elastography values of children with and without liver disease have been identified [36,37] whereas others found no effect of age [38]. We did not find age to significantly change the odds for advanced fibrosis.

Severe hepatic inflammatory activity has been shown to increase liver stiffness measurements with potential overestimation of noninvasive fibrosis staging [39,40]. Consequently elastography should be cautiously interpreted in patients with elevated alanine aminotransferase levels [22]. To account for this, we included alanine aminotransferase in our models and found that this interaction had the expexted effect. For lower levels the effect of the liver stiffness measures were significantly increased in all models compared to patients with high alanine aminotransferase (>470 U/L). Accordingly, patients with high alanine aminotransferaselevels were also excluded from the final nomograms to increase their accuracy since a high liver stiffness measure in these patients likely reflects inflammation rather than fibrotic transformation.

The effects of all included elastographic measures were significantly altered in models combining two or more modalities, with no relevant increase in discriminatory ability. This observation could be ascribed to a very high degree of correlation between the measures and hence considerable collinearity [41].

Ideally, precise noninvasive differentiation between all stages of fibrosis is desirable in assessment of pediatric liver disease. However, substantial overlap of elastography values, most notably in low fibrotic stages, is well described in both children and adults, independent of modality of choice [9,14,35,42,43]. In fibrotic transformation, deposition of excessive collagen and extracellular matrix proteins correlate with increased fibrosis stage in a curvilinear, rather than linear, way. Differences in hepatic collagen content have shown substantial overlap across fibrosis stages, with the greatest differences in higher fibrotic stages [44]. This contributes likely to lower discriminatory values of elastography in mild fibrosis, since detectable changes in collagen deposition and stiffness are minor until a certain threshold is reached. Hence, repeatedly decreased accuracies have been described in lower fibrotic stages, and reservations should be taken upon interpretation of liver stiffness values in terms of exact fibrosis stage [9,14].

TE and MRe were chosen for the final models. TE based on a high discriminatory accuracy, applicability, low costs, and potential for being used widely in the clinic across age groups. MRe presenting high discriminatory accuracy, significantly lower failure rate and evaluation of a larger proportion of the hepatic tissue, may possess important advantages in certain patients, including severe obesity, ascites and liver transplantation [19]. Importantly MR imaging is a cornerstone modality in the work-up of children with liver disease. With a limited extra time spent on additional sequences MR provides valuable information on hepatic anatomy and pathology including liver tissue contour, local inflammation, focal lesions, heterogeneous enlargement along with information on vascular and biliary tree autonomy, all off which may prove essential for diagnosis, treatment and follow-up of patients with pediatric liver disease. We present nomograms for the models, as a complementary decision tool to aid clinicians in noninvasive risk stratification of patients in terms of presenting advanced fibrosis. Based on the costs and need for anesthesia in younger children, our results do not suggest an additional role of MRe in routine risk stratification of advanced fibrosis in all patients at present, and further research is needed to clarify the role of MRe in pediatric liver disease. However, in children undergoing MR cholangiopancreatography and other abdominal MR imaging, MRe should, in our opinion, be considered an important supplement for fibrosis assessment. Incorporating B-mode examination and flow evaluation USe may be helpful in patients needing additional hepatobiliary imaging, however we did not find USe to provide additional information in terms of the risk for advanced fibrosis.

A number of considerations were made to optimize reproducibility and external validity of our results. Based on expert group consensus, outcome and covariates were a priori defined. Acknowledging the lack of knowledge on the velocity of fibrotic transformation, only elastography exams performed within 3 months of biopsy were included, as recommended in adults [45], and the majority of exams were performed within a minimal time interval between elastography and biopsy (median of 0–1 days). Time intervals of 6–12 months from biopsy are often reported in performance studies of elastography [14,31,43,46], which undoubtedly increases the risk of interim progression or regression of disease.

Pediatric studies involving multiple modalities, extensive planning and dependence on cooperation of the child, are susceptible to missing data which might bias estimated effects [47]. We conducted multiple imputations to account for, and to assess a potential bias of missing data. We found comparable results in complete case and multiple imputation analyses, indicating no bias induced by missing covariates. However, increased variances of the estimates were evident due to the imputations.

Our study has certain important limitations. First, we contribute knowledge from a single center. A predictive model will perform better in the dataset upon which it is based, and hence our findings need validation in other settings, preferably multicenter studies. Given the heterogeneity of included patients, firm conclusions on specific diagnoses could not be drawn. However, this heterogeneity represents the everyday scenario for pediatric hepatologists, and pediatric liver disease constitutes a range of disorders with a rare occurrence, but a similar need for precise estimation of disease severity. Importantly we show, that elastography measures predict advanced fibrosis despite differences in age, and our results confirm the findings of more uniform cohorts of pediatric patients.

Interestingly, we originally considered including extrahepatic cholestasis observed on hepatobiliary ultrasonography as a covariate, since this has been reported to affect elastography [48]. However only three patients presented ultrasonic findings suggesting cholestasis, and hence, this was not included in the model. Other factors including body mass index, necrosis and hepatic congestion have been proposed as factors potentially affecting measured liver stiffness [8]. However, we restricted our model to three predefined covariates of expected clinical importance and focused on an applicable and potentially widely used model. Further studies are needed to confirm potential roles of additional factors.

Ideally a gold standard examination presents a sensitivity and a specificity of 100% for discriminating the outcome of interest [49]. Histopathologic fibrosis grading of liver biopsies as a gold standard possess markedly lower accuracies, due to sampling variability, heterogenous distribution of fibrosis and interobserver variability among others [50]. This was evidenced by a disagreement rate of 11.1% among two experienced hepatopathologists in the present study, most notably in mild fibrosis. Hence, liver biopsy as the reference standard presents inherent limitations, and rigorous evaluation even among experienced hepatopathologists should be encouraged. Comparison of continuous elastography results with ordinally categorized histologic fibrosis stages, along with some degree of uncertainty in biopsy fibrosis staging, may prevent perfect accordance. This should be recognized upon interpretation of noninvasive measures of hepatic fibrosis.

## 5. Conclusions

In conclusion both TE, MRe and USe show high discriminatory values for predicting advanced fibrosis in a single center cohort of pediatric patients with various liver diseases. TE and MRe displayed the highest discriminatory abilities although not significantly different from USe. No significant increase in discriminatory ability for advanced fibrosis was seen upon combination of different elastographic techniques.

## Figures and Tables

**Table 1:** Characteristics of included patients.

	Patients (*n* = 93)	
Clinical characteristics		
Sex (*n* = male/female)	48/45	
Listed for Liver transplantation, *n* (%)	6	(6.5%)
Anthropometrics, median (range)		
Age (years)	12	(0.06–19.6)
Height (cm)	151	(41–192.5)
Weight (kg)	41	(1.9–115)
Body Mass Index	18	(11.3–43.8)
Etiology, *n* (%)		
Previous liver transplantation	25	(26.9%)
Autoimmune hepatitis without primary sclerosing cholangitis	17	(18.3%)
Autoimmune hepatitis with primary sclerosing cholangitis	10	(10.8%)
Biliary atresia	10	(10.8%)
Hepatitis B/C	3	(3.2%)
Polycystic liver and kidney disease	3	(3.2%)
Non-alcoholic fatty liver disease	2	(2.2%)
Alfa-1 antitrypsin deficiency	3	(3.2%)
Wilson Disease	3	(3.2%)
Acute liver failure	2	(2.2%)
Alagille Syndrome	1	(1.1%)
Primary sclerosing cholangitis	1	(1.1%)
Portal vein thrombosis	1	(1.1%)
Short bowel syndrome	1	(1.1%)
Crigler Najjar Syndrome	1	(1.1%)
Other ^a^	4	(4.3%)
Elevated transaminases of unknown origin	4	(4.3%)
Cholestasis of unknown origin	2	(2.2%)
Blood Biochemistry, median (range)		
Alanine aminotransferase (IU/L)	83	(6–2190)
Aspartate aminotransferase (IU/L)	66	(17–977)
Alkaline phosphatase (IU/L)	265	(41–1300)
Gamma-glutamyl transferase (IU/L)	62	(11–954)
Bilirubin (µmol/L)	11	(3–433)
Albumin (g/L)	37	(21–49)
International normalized ratio	1.2	(0.9–4.9)
Plasma coagulation factors II, VII, X (U/L)	0.7	(0.1–1.2)
Platelets (×10^9^/L)	240	(27–558)
Hemoglobin (mmol/L)	7.9	(4.9–10.1)
Leucocytes (×10^9^/L)	6.3	(1.4–33.5)
Creatinine (µmol/L)	48	(11–440)
Urea (mmol/L)	3.8	(0.8–25.8)
Sodium (mmol/L)	140	(132–146)
Potassium (mmol/L)	3.9	(2.7–5.2)
Magnesium (mmol/L)	0.80	(0.53–1.02)
Phosphate (mmol/L)	1.37	(0.83–2.05)

^a^ Prematurity/Immaturity with or without necrotizing enterocolitis (*n* = 2), Celiac Disease (*n* = 1), Epstein-Barr virus (*n* = 1).

**Table 2:** Results from multivariate logistic regression models with advanced fibrosis (F3-4) as outcome and liver stiffness measures age and Alanine aminotransferase levels ≤ 470 as covariates.

Model	OR Elastography (CI)		OR Age (CI)		Area under Curve (CI)		Brier Score (CI)	
Complete Case								
USe Use (age ≥ 5)	602	(20.30–17,900)	1.04	(0.94–1.16)	0.798 0.784	(0.661–0.935) (0.607–0.960)	0.115 0.121	(0.064–0.166) (0.062–0.181)
TE_log2_ TE_log2_ (age ≥ 5)	4.96	(2.35–10.50)	0.95	(0.80–1.14)	0.862 0.878	(0.758–0.966) (0.781–0.976)	0.118 0.104	(0.065–0.171) (0.054–0.155)
MRe_log2_ (age ≥ 5)	233	(11.12–4873)	0.82	(0.58–1.16)	0.896	(0.798–0.994)	0.098	(0.049–0.148)
Multiple imputation								
USe USe (age ≥ 5)	1155	(9.39–142,000)	1.04	(0.94–1.16)	0.786 0.765		0.115 0.121	
TE_log2_ TE_log2_ (age ≥ 5)	5.30	(2.18–12.90)	1.04	(0.90–1.19)	0.834 0.847		0.117 0.111	
MRe_log2_ (age ≥ 5)	305	(13.10–7100)	0.86	(0.64–1.15)	0.898		0.103	

USe: 2D shear wave ultrasound elastography; TE: Transient elastography; MRe: Magnetic Resonance elastography.

**Table 3:** Results from multivariate logistic regression models with advanced fibrosis (F3-4) as outcome and combination of liver stiffness measures age and Alanine aminotransferase levels ≤ 470 as covariates.

Model	OR (CI)		Area under Curve	Brier Score
Complete case all (age ≥ 5)			0.956	0.068
USe	335	(0.183–613,000)		
TE	0.50	(0.029–8.41)		
MRe	82	(0.076–88,500)		
Age	1.14	(0.624–2.09)		
Complete case TE/MRe (age ≥ 5)			0.881	0.096
TE	5.80	(0.630–53.40)		
MRe	3.14	(0.104–95.30)		
Age	0.78	(0.54–1.13)		
Multiple imputation all (age ≥ 5)			0.925	0.082
USe	1.37	(0.00–3551)		
TE	5.33	(0.24–1465)		
MRe	5.25	(0.00–373)		
Age	0.81	(0.54–1.44)		
Multiple imputation TE/MRe (age ≥ 5)			0.879	0.103
TE	6.69	(0.0584–76.8)		
MRe	2.94	(0.031–276)		
Age	0.82	(0.615–1.09)		

USe: 2D shear wave ultrasound elastography; TE: Transient elastography; MRe: Magnetic Resonance elastography.

## Data Availability

Study data is stored on a logged and secured regional drive according to rules from the Danish Data Protection authority.
